# Suicidal Ideation in the Setting of Neurocysticercosis

**DOI:** 10.7759/cureus.53934

**Published:** 2024-02-09

**Authors:** Jake Smith, Diana Anand, Sachidanand Peteru, Hannah Lynch, Jashandeep Kaur

**Affiliations:** 1 Psychiatry, Jamaica Hospital Medical Center, New York City, USA; 2 Consultation-Liaison Psychiatry, Flushing Hospital Medical Center, New York City, USA; 3 Psychiatry, New York Institute of Technology College of Osteopathic Medicine, New York City, USA

**Keywords:** pork tapeworm, taenia solium, seizure, suicide, depression, neurocysticercosis

## Abstract

Neurocysticercosis (NCC) is considered a significant health concern in developing countries in parts of Asia, Africa, and Central and South America. However, with the increased immigration, it is now becoming increasingly prevalent in the United States. NCC has psychiatric implications often neglected and not recognized in the initial diagnostic workup of patients from developing countries suffering from seizures and psychiatric illnesses, such as depression. This case report aims to signify the presentation of NCC and illustrate the importance of the psychiatric manifestations of NCC in patients. We discuss the case of a 32-year-old female patient from a rural town in Central America who immigrated to New York and presented with uncontrolled seizures and symptomatic depression with suicidal ideations.

## Introduction

Neurocysticercosis (NCC) is a helminthic infection of the nervous system that results from the ingestion of eggs from *Taenia solium*, an adult tapeworm [1,2]. It is a common parasitic infection that is the cause of a public health problem affecting impoverished rural populations with inadequate sanitation, including in Latin America [3]. However, due to the rising immigration of Latin Americans to the United States, NCC is now prevalent in the United States, especially in the southwestern states [2].

The symptoms of NCC mimic psychiatric and neurological disorders in up to 15% of infected patients, including affective disorder, schizophrenia, depression, headaches, seizures, and epilepsy [4]. Annually, there are more than 2,300 admissions due to NCC in the United States, and only 2% of seizure cases admitted are discovered to be attributed to NCC [5]. We found very few publications that consider NCC's psychiatric symptoms; however, studies have shown that patients with NCC show a decline in the quality of life, depression, and moderate cognitive impairment [4]. It has been suggested, by a prior study on the quality of life in patients with NCC, that the seizures and epilepsy caused by NCC affect the psychosocial aspects of life, leading to depression and anxiety [6]. The above situation creates a concern for physicians unaware of the epidemiological importance of NCC and its effect on psychiatric issues.

## Case presentation

A 32-year-old Hispanic female with a past medical history of seizures and a past psychiatric history of major depressive disorder presented to the emergency department on account of suicidal ideations; however, she denied any specific plan. She stated that the suicidal ideations were due to the financial stress caused by the persistent seizures she had been experiencing that prevented her from stable employment. The seizures had started when she was 18 years old in her home country, Guatemala, and occurred at least two times per week. She reported multiple evaluations by many neurologists in multiple hospitals, and her treatment included levetiracetam. However, the frequency of her seizures remained the same despite medication compliance. Due to the socioeconomic effects of the seizures on the patient, she endorsed symptoms of depressed mood, anhedonia, and feelings of hopelessness and helplessness with suicidal ideations. She also reported a previous suicide attempt via prescription drug overdose two years before presentation when the patient's seizures were poorly controlled and more frequent.

In the emergency department, a psychiatry consultation completed an evaluation for her depressive symptoms and a neurologist for her seizures. Neurology recommended discontinuing levetiracetam due to poor efficacy and further managing her seizures to outpatient. The patient was medically cleared and subsequently admitted to the inpatient psychiatric unit to treat her depression. 

During the hospital course, the patient was started on sertraline 50 mg daily to manage her depression. Upon further evaluation, the patient admitted to non-compliance with her anti-seizure medications because the patient stated that the medication caused sedation and she had a newborn child who needed her to be awake. Neurology was consulted again for additional insight into her medical disorder. The patient described her seizures as generalized tonic-clonic with a confused postictal state. Her last seizure was three days prior to her admission. She denied urinary or fecal incontinence.

Upon neurological exam, the patient was alert and oriented with intact motor and sensory skills. Her labs indicated a mild elevation in her liver enzymes with alanine transaminase at 41 U/L (reference range: 0-34 U/L) and aspartate transaminase at 39U/L (reference range: 14-36 U/L), as well as elevated eosinophils at 6.3% (reference range: 0-3%). Neurology recommended avoiding levetiracetam due to its potential adverse effects on liver function. It recommended lamotrigine 25 mg twice a day for one week, and then increasing the daily dose by 25 mg weekly until it reaches the maximum dose of 100 mg a day. Neurology also recommended starting lacosamide 100 mg twice daily with titration of lamotrigine and discontinuing lacosamide once the lamotrigine dose was 100 mg daily. 

**Figure 1:**
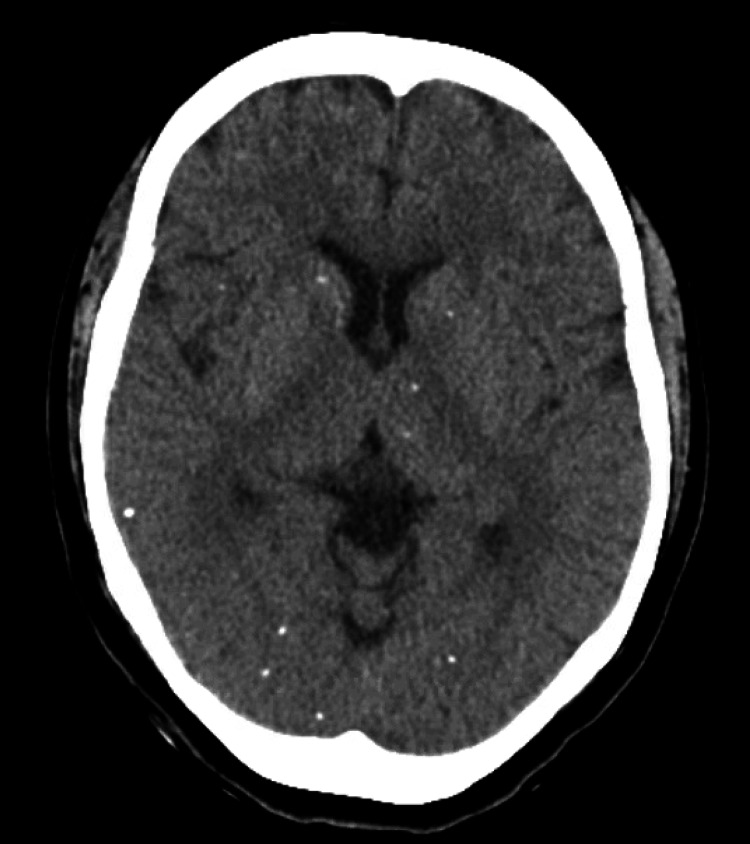
CT Head showing multiple scattered calcifications throughout the white matter.

**Figure 2:**
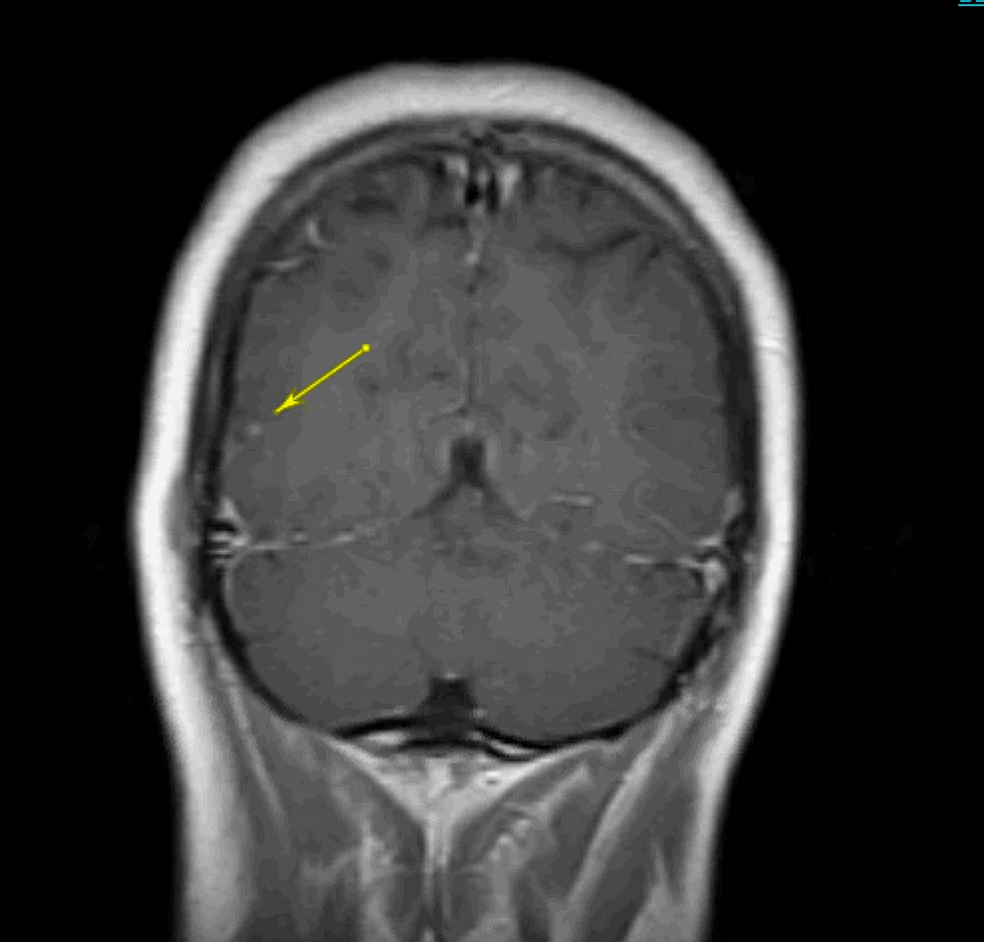
MRI Brain showing a tiny focus of enhancement in the right posterior temporal, parietal, and occipital areas.

CT head showed multiple scattered calcifications throughout the white matter consistent with chronic neurocysticercosis (Figure 1). Gadolinium-based MRI showed a tiny focus of enhancement in the right posterior temporal, parietal, and occipital areas in the brain parenchyma (Figure 2). Due to the above findings, following the infectious disease consultation, the patient started albendazole 15 mg/kg every 12 hours for 10 days while awaiting results from cysticerci serology.

## Discussion

NCC is a parasitic brain infection due to *T. solium*, a pork tapeworm. In endemic regions, this infection is common. If humans ingest the larvae through uncooked pork meat sources, the tapeworm can grow and reside within the intestines, which is called taeniasis. However, humans can ingest the *T. solium* cysts through fecal contamination, and these eggs will hatch within the body and distribute to the CNS, resulting in NCC. Hosts that carry the tapeworm can also become infected with the tapeworm eggs themselves, but this is a less likely route [7]. 

*T. solium* cysts can be intraparenchymal or extra parenchymal. If intraparenchymal, like in our patient, the cysts will cause an inflammatory response in the host. The large cysts cause mass effects. Intraparenchymal cysts are usually fluid-filled and may degenerate or calcify, which represents dead larvae. In the case of extra parenchymal, the cysts will reside in the ventricular cavities, which may block the CSF circulation due to mass effect, leading to hydrocephalus and intracranial hypertension [7]. 

The presentation of NCC is variable and depends on the location of the *T. solium* cysts. Patients can be asymptomatic initially, while the parasite starts in the intestines and then hatches and spreads. The host's immune response may destroy the parasite before it spreads to the CNS, or it can stay dormant in the brain for years [7]. The hallmark symptom of NCC is seizure [5]. Seizures are due to inflammation and neuronal damage from the cysts [7] or due to degenerating intraparenchymal cysts [5]. The other common symptoms include headache, intracranial hypertension, confusion, and focal neurologic signs. 

NCC is a clinical diagnosis from clinical manifestations, a history of endemic areas, and imaging results. CT and MRI are the imaging methods of choice since they can fully characterize cysts. MRI is best for small cysts and can provide information on inflammation. CT is better for calcified lesions and intraparenchymal cysts [7]. On imaging, cysts will likely have focal contrast enhancement, well-defined borders, contain a liquid within the borders, and may or may not have edema and signs of inflammation around the lesions [5,7]. If imaging is inconclusive, then NCC serology can be obtained, like lentil-lectin purified antigens (LLGP) on enzyme-linked immuno electrotransfer blot (EITB) antibody detection [7]. 

Treatment involves controlling the seizures, intracranial hypertension, and antiparasitic treatment to destroy the cysts. Albendazole is the antiparasitic drug of choice [7]. Treatment with albendazole is generally for one to two weeks at 15 mg/kg/day [5]. With the destruction of all the cysts, it is common for patients to experience fewer seizures and neurologic symptoms, especially if cysts are intraparenchymal [8]. 

Psychiatric manifestations and disorders secondary to NCC have not been a large area of research. There have been reports of up to 15% of patients with NCC developing various psychiatric illnesses, including depression, anxiety, and personality changes. There is no correlation with the extent or length of NCC in the patient, and regardless of NCC prognosis, patients suffer from depression more so than the average population. Depression is the most common comorbid psychiatric disorder and then mixed anxiety depression. There are no reported cases of suicidal ideation or attempts in NCC. The pathophysiology of the psychiatric manifestations of NCC is currently unknown [4]. 

Our patient presented to the ED with a breakthrough seizure and suicidal ideation with a history of frequent weekly seizures for 18 years. The patient also reported a significant psychiatric history of depression beginning around the same time as when seizures began, with a previous suicide attempt two years prior to presentation. Upon evaluation, our patient attributed her chronic depression to how the seizures have affected her life, like being scared to hold her infant daughter in fear of dropping the child if she had a seizure. To our knowledge, there have been no reports in the literature of suicidal ideation or suicide attempts in the setting of NCC. This case is important because it highlights the need to consider the psychiatric associations with NCC, particularly suicidal ideation and suicide attempts. It also demonstrates the importance of a broad differential when a patient presents with symptoms of depression alongside a history of seizures. 

## Conclusions

This patient presented to the ED with suicidal ideation and a history of recurrent, poorly controlled seizures. CT head determined the NCC diagnosis, a known cause of seizures. Our case supports the risk of an association of NCC with depression, making the patient vulnerable to suicidal ideas or behaviors as a complication. It is important in such a case to explore various differential diagnoses, including adjustment disorder with depressed mood, to ensure the patient receives appropriate treatment. Given the severity of the patient's depressive symptoms accompanied by her persistent suicidality for more than six months, the patient met the criteria for severe major depression disorder. Although New York is not an endemic region for NCC, our case demonstrates the importance of keeping a broad differential and ruling out medical diagnoses when evaluating psychiatric disorders. 
